# TALEN-mediated homologous-recombination-based fibroin light chain in-fusion expression system in *Bombyx mori*

**DOI:** 10.3389/fbioe.2024.1399629

**Published:** 2024-05-20

**Authors:** Shihua Yu, Huoqing Zheng, Xiaogang Ye, Xiangping Dai, Xinqiu Wang, Shuo Zhao, Xiaoyan Dai, Boxiong Zhong

**Affiliations:** ^1^ College of Animal Sciences, Zhejiang University, Hangzhou, China; ^2^ Key Laboratory of Silkworm and Bee Resource Utilization and Innovation of Zhejiang Province, Hangzhou, China; ^3^ Department of Laboratory Medicine, The First Affiliated Hospital of Henan University of Chinese Medicine, Zheng Zhou, China; ^4^ Suposik Bioscience Technologies Ltd., Jiaxing, China

**Keywords:** TALEN, gene editing, molecular docking, FIBL, *Bombyx mori*

## Abstract

Silkworm was the first domesticated insect and has important economic value. It has also become an ideal model organism with applications in genetic and expression studies. In recent years, the use of transgenic strategies has made the silkworm silk gland an attractive bioreactor for the production of recombinant proteins, in particular, *piggyBac*-mediated transgenes. However, owing to differences in regulatory elements such as promoters, the expression levels of exogenous proteins have not reached expectations. Here, we used targeted gene editing to achieve site-specific integration of exogenous genes on genomic DNA and established the fibroin light chain (FibL) in-fusion expression system by TALEN-mediated homology-directed recombination. First, the histidine-rich cuticular protein (CP) was successfully site-directed inserted into the native FibL, and the FibL–CP fusion gene was correctly transcribed and expressed in the posterior silk gland under the control of the endogenous FibL promoter, with a protein expression level comparable with that of the native FibL protein. Moreover, we showed based on molecular docking that the fusion of FibL with cuticular protein may have a negative effect on disulfide bond formation between the C-terminal domain of fibroin heavy chain (FibH) and FibL–CP, resulting in abnormal spinning and cocoon in homozygotes, indicating a significant role of FibL in silk protein formation and secretion. Our results demonstrate the feasibility of using the FibL fusion system to express exogenous proteins in silkworm. We expect that this bioreactor system will be used to produce more proteins of interest, expanding the application value of the silk gland bioreactor.

## 1 Introduction

The silkworm has an economic significance owing to its high capacity for protein synthesis, as well as the advantages of a short growth cycle, suitability for large-scale breeding, and lack of disease transmission to humans. The silk gland is the only tissue of the silkworm that can synthesize and secrete silk proteins with high efficiency and specificity. The development of DNA recombinant technology has enabled research on the production of recombinant proteins using the silkworm silk gland (Nawa et al., 1971; Ninaki, 1985). In 2000, Tamura et al. made the first transgenic silkworm based on the *piggyBac* transposon (Tamura et al., 2000); this represented the establishment of silkworm transgenic technology. Subsequently, targeted gene editing systems such as zinc-finger nucleases (ZNFs), transcription activator-like effector nucleases (TALEN), and clustered regularly interspaced short palindromic repeats Cas9 nucleases (CRISPR/Cas9) have been applied for precise knockout or knock-in of the silkworm genome. These genome editing systems have greatly facilitated gene function studies, genetic improvements, and the development of silk gland bioreactors in silkworm.

Silk proteins are mainly composed of silk fibroin and sericin; silk fibroin contains fibroin heavy chain (FibH), fibroin light chain (FibL), and fibrohexamerin (P25) (Inoue et al., 2000); and sericin contains sericin1–3 (ser1, ser2, and ser3) and the newly reported sericin4–5 (ser4 and ser5) (Guo et al., 2022). Expression systems based on FibH, FibL, and ser1 promoters were often used because they could express relatively high levels of exogenous proteins (Xu, 2014). Since 2003, when the FibL promoter was first used to specifically express recombinant proteins in silkworm silk glands via *piggyBac* transposon-mediated transgenes (Tomita et al., 2003), the production of recombinant proteins using transgenic silkworm silk gland bioreactors has entered a period of rapid development (Long et al., 2021; Wu et al., 2021; Chen et al., 2022). In addition, modification of *piggyBac* transposon-based transgene regulatory elements has been found to improve the expression levels of exogenous proteins (Iizuka et al., 2008; Zhao et al., 2021), and a recent study showed that the use of native regulatory elements could increase the production of exogenous proteins up to 15-fold compared with an artificially truncated promoter (Li et al., 2021). Therefore, the use of endogenous regulatory elements is an ideal method to improve the expression levels of exogenous proteins.

Although the *piggyBac* transposon-mediated transgenic silkworm silk gland has been gradually developed as a bioreactor in recent years (Tomita et al., 2003; Long et al., 2021; Wu et al., 2021), *piggyBac*-mediated transgenes have some limitations such as uncertain insertion sites, low expression, and gene drift (Fraser, 2012). However, the advent of targeted gene editing, which creates double-stranded breaks (DSBs) at genomic DNA, allows researchers to modify specific locations in the genome of the organism of interest; it can also enable more precise gene modification and more stable inheritance. Importantly, it allows the transcription and translation of exogenous genes under the endogenous regulatory elements of the organism. In these systems, the efficiency of targeted gene editing mediated by ZNFs and CRISPR/Cas9 was low (Takasu et al., 2010; Junpeng et al., 2023); only TALEN-mediated gene transformation efficiency was comparable with that of transposon-mediated transgenes (Xu et al., 2018; Li et al., 2021; Takasu et al., 2023). Moreover, FibH is one of the main silk proteins, and the expression of exogenous proteins using the native FibH promoter has been shown to have an impact on spinning and cocoon (Xu et al., 2018; Takasu et al., 2023). Here, we fused a histidine-rich cuticular protein (Gene ID: 101743422, CP) (Futahashi et al., 2008), which has been reported to have potential as a biological film material (Liu et al., 2021), to native FibL by TALEN-mediated homology-directed recombination (HDR). In the FibL in-fusion expression system, CP and FibL were successfully fused and correctly transcribed and translated in the posterior silk gland (PSG) of the transgenic silkworm, and western blotting analysis showed that the expression level of FibL–CP protein was comparable with that of the native FibL protein. Thus, this study establishes an endogenous FibL expression system in silkworm, which has the potential to produce more valuable recombinant proteins in silk gland bioreactors in the future.

## 2 Materials and methods

### 2.1 Design of TALEN target sites and construction of TALEN vector

Arrays of TAL effector nucleases targeting the silkworm genome were designed using TAL Effector Nucleotide Targeter 2.0 (https://tale-nt.cac.cornell.edu/) (Doyle et al., 2012). The construction of TALEN vectors were performed by Golden-Gate assembly according to the instruction provided by the manufacturer of the Golden Gate TALEN and TAL effector kit 2.0 (Addgene number: 1000000024) (Cermak et al., 2011). All plasmids were verified by Sanger sequencing.

### 2.2 Homologous recombination (HR)-mediated donor construction

The HR donor contained a left homology arm and right homology arm amplified and sequenced from the silkworm genome; the TALEN target site was placed at the 5′end of the left arm and the 3′end of the right arm. The CP coding sequence was seamlessly designed behind the left arm with the poly(A) sequence of FibH. Those above fragments were synthesized and cloned into the pUC-GW-Amp vector (GENEWIZ) to generate a pUC-L-CP-R plasmid. The HR3-IE1-EGFP-SV40 cassette was cut from the pBac[IE1-EGFP]-2×PySp1-A.arg plasmid (stored in our laboratory) using AflⅡ restriction endonuclease and cloned into the pUC-L-CP-R vector to generate a pUC-FibL–CP–EGFP HR-mediated donor. The donor vector was verified by Sanger sequencing.

### 2.3 Purification and microinjection of TALEN mRNA and donor

TALEN vectors were linearized by XbaⅠ restriction endonuclease digestion and purified with phenol/chloroform/isoamylalcohol (25:24:1); then, TALEN mRNAs were synthesized *in vitro* with an mMESSAGE mMACHINE™ T7 Kit (Invitrogen), respectively. The donor vector and TALEN mRNAs were purified with phenol/chloroform/isoamylalcohol (25:24:1) and dissolved in 1× phosphate-buffered saline (1×PBS buffer). TALEN mRNA (300 ng/μL) and the donor vector (200 ng/μL) were mixed and injected into *Lan10* silkworm embryos within 8 h after oviposition. The injected eggs were incubated at 25 °C, 90% humidity, until hatching. The G0 larvae were reared with fresh mulberry leaves until the cocoon stage. G0 moths were mated with wild-type (WT) individuals, and positive larvae in G1 were screened under a green fluorescence microscope (Olympus, Tokyo, Japan).

### 2.4 Gene identification of positive individuals

Genomic DNA was extracted from the PSGs of positive and WT individuals using an Ezup Column Animal Genomic DNA Purification Kit (Sangon Biotech), and the 5′and 3′junction were confirmed by PCR amplification. Sanger sequencing was performed directly on the amplification products. Quantitative real-time PCR (qRT-PCR) analysis was performed as previously described (You et al., 2018). Briefly, total RNA was extracted from PSGs of positive and WT individuals on the third day of the fifth instar larvae using TRIzol reagent (Invitrogen). The cDNA was synthesized using HiScript III RT SuperMix for qPCR (+ gDNA wiper) (Vazyme). Relative transcript levels of FibL and FibL–CP were determined by qRT-PCR using ChamQ Universal SYBR qPCR Master Mix (Vazyme). The PCR reaction was performed according to the manufacturer’s instructions, all of the primers used are listed in the (Supplementary Table S1), and three independent replicates of each sample were used.

### 2.5 Western blotting analysis

Proteins were extracted according to a previously described method with some modifications (Wu et al., 2021). Briefly, silk gland and cocoon shell proteins were extracted with sodium dodecyl sulfate (SDS) buffer (1:40, wt/vol) containing 5% (vol/vol) β-mercaptoethanol, and the supernatants were collected by centrifuging after incubation at 37 °C for 8 h. The extracted proteins were quantified using a Modified BCA Protein Assay Kit (Sangon Biotech); then, western blotting analysis was performed. FibL and FibL–CP proteins were detected using FibL polyclonal antibodies as primary antibodies (1:3,000 dilution, GenScript), and horseradish-peroxidase-conjugated goat anti-rabbit IgG was used as the secondary antibody (1:5,000 dilution, Sangon Biotech). The signal was detected using highly sensitive Plus ECL Reagent (Sangon Biotech). ImageJ software was used to quantify band intensities.

### 2.6 Evaluation of mechanical properties of silk fibers

Ten cocoons were randomly chosen from each of the WT and FibL–CP strains, and three pieces of silk were drawn from each cocoon and tested. Silk fibers were prepared and tested as described previously (Perez-Rigueiro et al., 2000; Zhao et al., 2007; Zhao et al., 2021). Briefly, the diameter of each silk fiber was measured using a three-dimensional digital microscope (Keyence), and the cross-sectional area of the silk was calculated as the average of five measurements. Tensile tests were performed on each silk fiber using an AGS-J Universal Test instrument (Shimadzu) with a cell load of 5 N. The toughness of the silk fiber was calculated by integrating the area under the stress–strain curves using mathematical software (Origin 2019b).

### 2.7 Structure prediction and molecular docking

The three-dimensional structure of the FibL monomer (AFDB accession: AF-P21828-F1) and the C-terminal domain (CTD) of Fib-H (AFDB accession: AF-Q1KS45-F1) were directly obtained from the AlphaFold Protein Structure Database, and the three-dimensional structure of FibL–CP was predicted using the I-TASSER online server (Yang and Zhang, 2015; Zheng et al., 2021; Zhou et al., 2022). Molecular docking of the CTD of FibH to the FibL and FibL–CP proteins, respectively, was performed using the HADDOCK online server (Wassenaar et al., 2012; van Zundert et al., 2016). The best docking results were visualized and analyzed using PyMOL software.

### 2.8 Statistical analysis

The experimental data were analyzed with Student’s t-test (*p < 0.05, **p < 0.01, ***p < 0.001, and n.s for *p* > 0.05). Correlation analysis and linear regression analysis were performed using Excel software. In the figures, error bars indicate the mean ± standard error of the mean.

## 3 Results

### 3.1 Construction of TALEN vector and verification of activity

**FIGURE 1:**
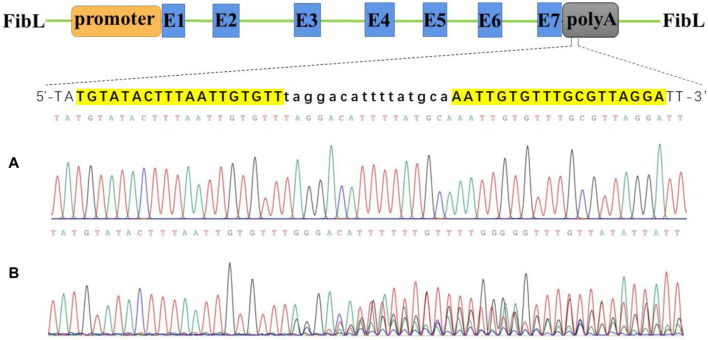
Activity test of TALEN targeted cutting of FibL by Sanger sequencing. **(A)** Wild type. **(B)** TALEN targeted cutting. FibL exons are represented by blue boxes and marked with E; TALEN-targeting sequences are listed separately, highlighted in yellow as recognition regions; and lowercase letters indicate spacer sequences.

We constructed a TAL effector by Golden-Gate assembly and ligated it into the VK006-06 vector (ViewSolid Biotech), which contains a T7 promoter for *in vitro* transcription. Two TALEN vectors containing different FOK Ⅰ enzyme form heterodimers to improve the efficiency of DNA cutting. In the 3′untranslated region of FibL, we designed two target sites. Three days after injection of TALEN mRNAs into silkworm eggs, the genomic DNA was extracted, and a DNA fragment near the targeting site was amplified for sequencing. One target site tested positive (Figure 1), whereas the other was inactive (Supplementary Figure S1). We used the positive target site for subsequent experiments.

### 3.2 Construction of FibL–CP in-fusion express transgenic silkworm

**FIGURE 2:**
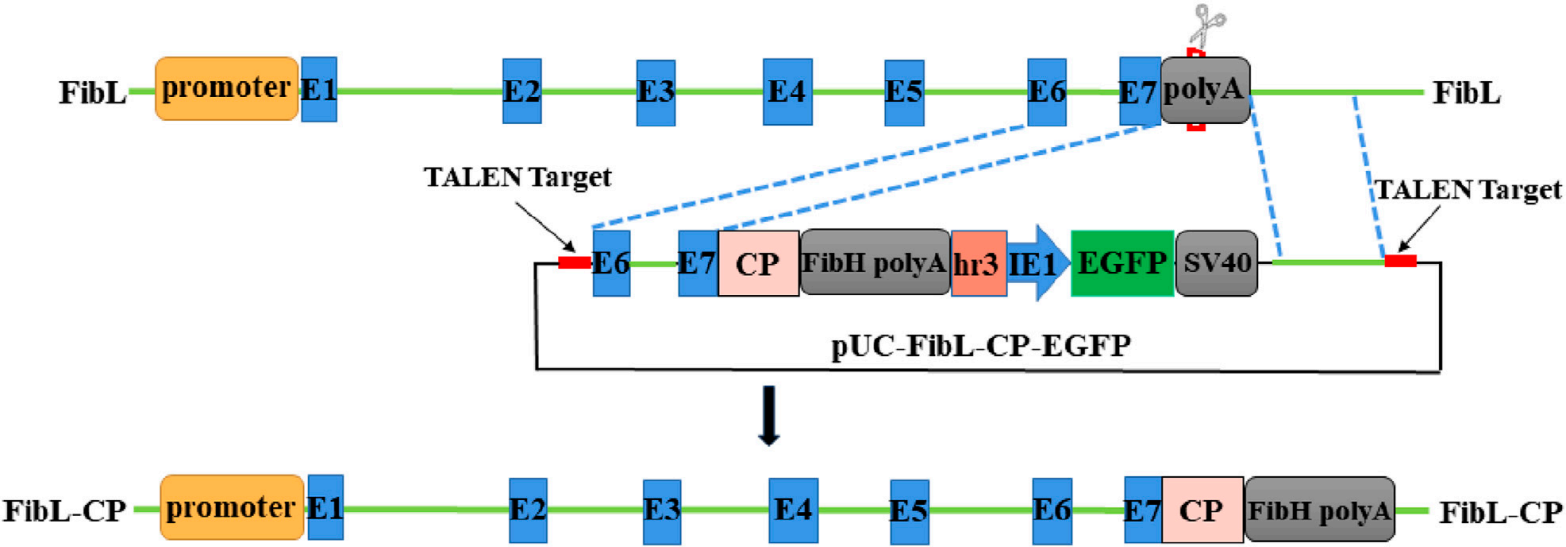
Schematic representation of FibL–CP fusion expression and donor vector. Green lines indicate genomic DNA sequences. The donor was designed to be cut by TALEN outside the 834-bp and 812-bp homology arms. *EGFP*, controlled by the baculovirus, IE1 promoter and HR3 enhancer, was used as a marker genes.

To achieve fusion expression of FibL and CP, we designed a donor vector containing the CP gene (Figure 2), and an 834-bp 5′-homologous arm and an 812-bp 3′-homologous arm were designed to facilitate HR. The CP gene was seamlessly inserted at the end of FibL to achieve FibL–CP fusion expression. Early studies have reported that linear DNA can improve the efficiency of HR (Wang et al., 2014; Takasu et al., 2016). Therefore, TALEN targeting sequences were added to both sides of the donor vector to achieve linearization *in vivo*. The TALEN mRNAs and donor plasmid were mixed and microinjected into silkworm eggs. Under the repair template we provided, HDR repair occurred, and the CP gene was site-directed inserted into FibL.

### 3.3 Selection and identification of FibL–CP fusion expression transgenic silkworm

**FIGURE 3:**
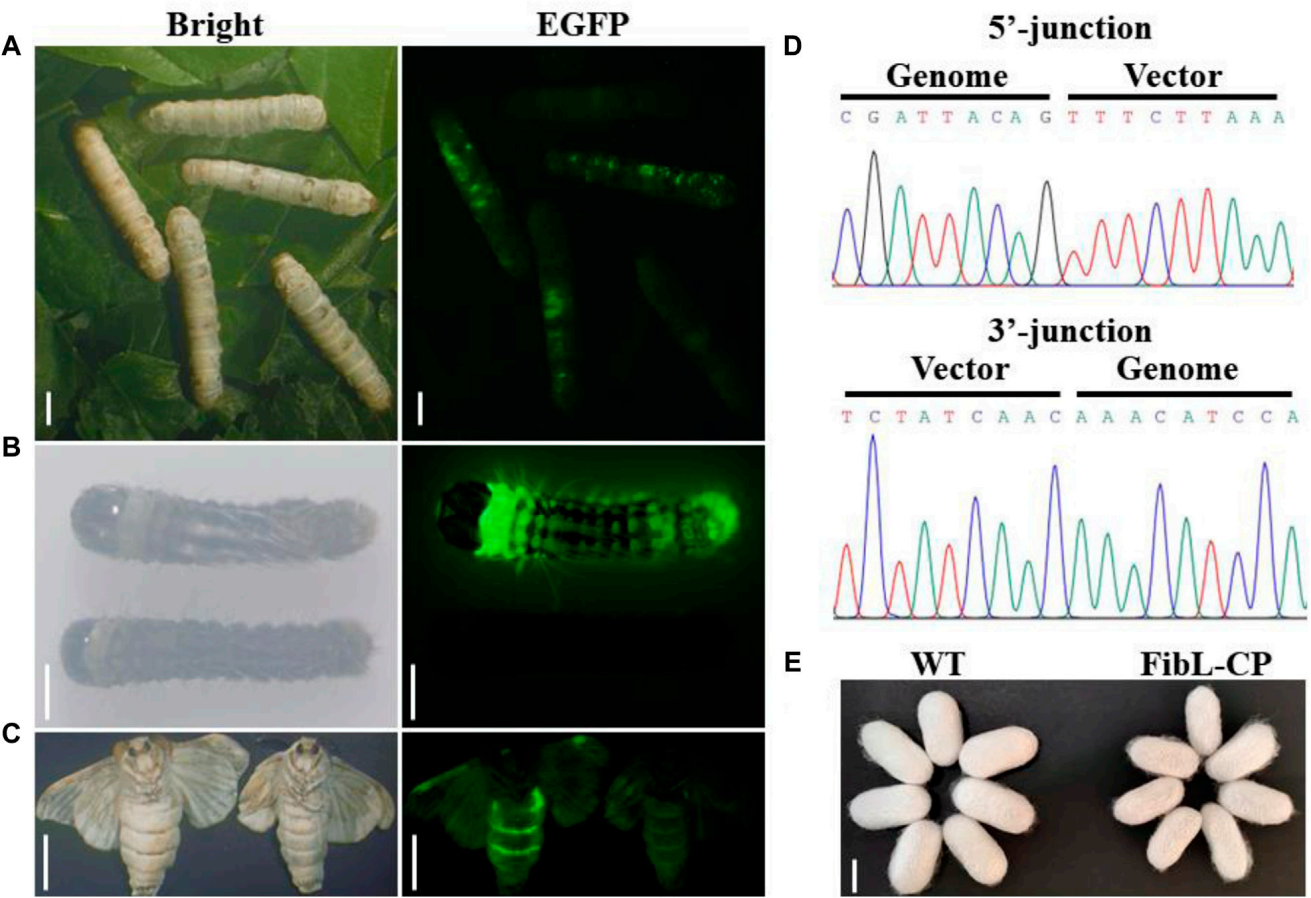
Screening and identification of G0-and G1-positive silkworms. Insects were photographed with normal light or under ultraviolet light with a GFP filter. **(A)** Fluorescent spot observation of G0 silkworm (scale bars: 5 mm). **(B)** Screening of positive silkworms on the first day of the first instar larvae of the G1 generation (scale bars: 2 mm). **(C)** Fluorescence observations of positive moths (scale bars: 9 mm). **(D)** Sequencing results to show 5′and 3′junction genome–donor integration. **(E)** Comparison of cocoon shell between FibL–CP and WT strains (scale bar: 15 mm).

**FIGURE 4:**
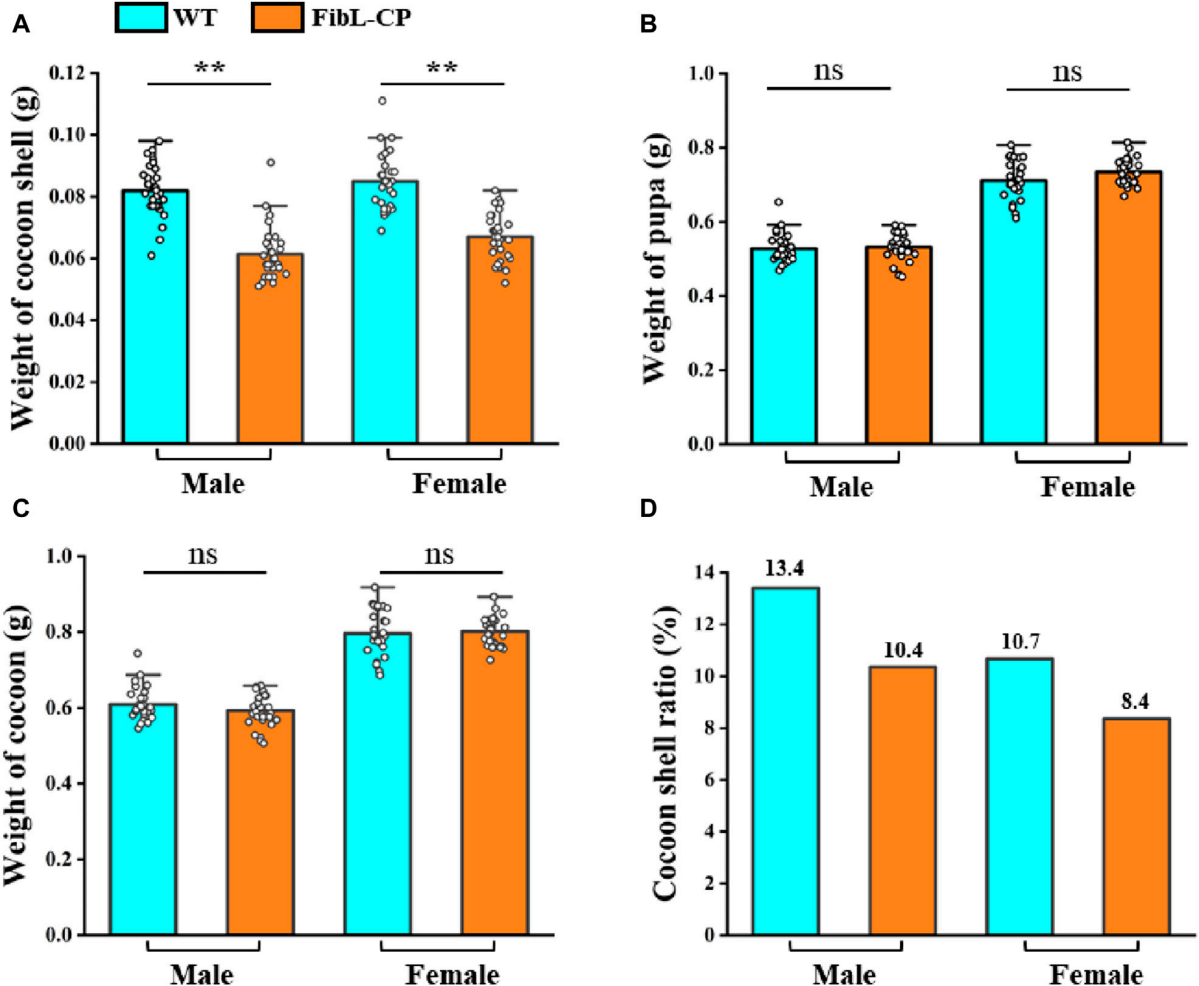
The statistical analysis of the weight of cocoon shell **(A)**, pupa **(B)**, cocoon **(C)** and cocoon shell ratio **(D)** (*n = 30*)

In the second instar larvae of G0, many lumps of fluorescence on the body surface of silkworm could be observed under the green fluorescence microscope (Figure 3A). G0 moths were crossed with WT, and positive larvae of the first instar of G1 were screened under a fluorescence microscope (Figure 3B). We screened six EGFP-positive G1 broods; the transformation efficiency was 9.2% (Table 1). Green fluorescence could be seen throughout the lifecycle (Figure 3C). Genomic DNA was extracted from mated G1-positive moths, and the 5′and 3′junctions were amplified. Sanger sequencing was performed directly on the amplification products; the results showed that the insertion was precise and seamless (Figure 3D and Supplementary Figure S2). The primers used for amplification are listed in Supplementary Table S1. The FibL–CP heterozygous animals showed successful spinning and cocooning, but the cocoon shells were a little smaller than those of the WT (Figure 3E). We weighed the cocoon shells of the FibL–CP and WT silkworms. In both females and males, the average cocoon shell weight of the FibL–CP silkworm was markedly decreased compared with that of the WT. However, the average weights of female and male pupae did not show obvious changes between the FibL–CP and WT silkworms (Figure 4). FibL–CP homozygotes showed abnormal spinning and cocooning (Supplementary Figure S3). These results indicate that insertion of the CP gene may affect the spinning of silkworm.

**TABLE 1:** Transformation efficiency of CP gene in FibL in-fusion expression system.

Vector	Injected	Hatched	G1 broods	Positive G1 broods	Transformation efficiency (%)
FibL–CP	700	118	65	6	9.2

### 3.4 Expression of FibL–CP fusion protein in FibL site-directed insertion system

**FIGURE 5:**
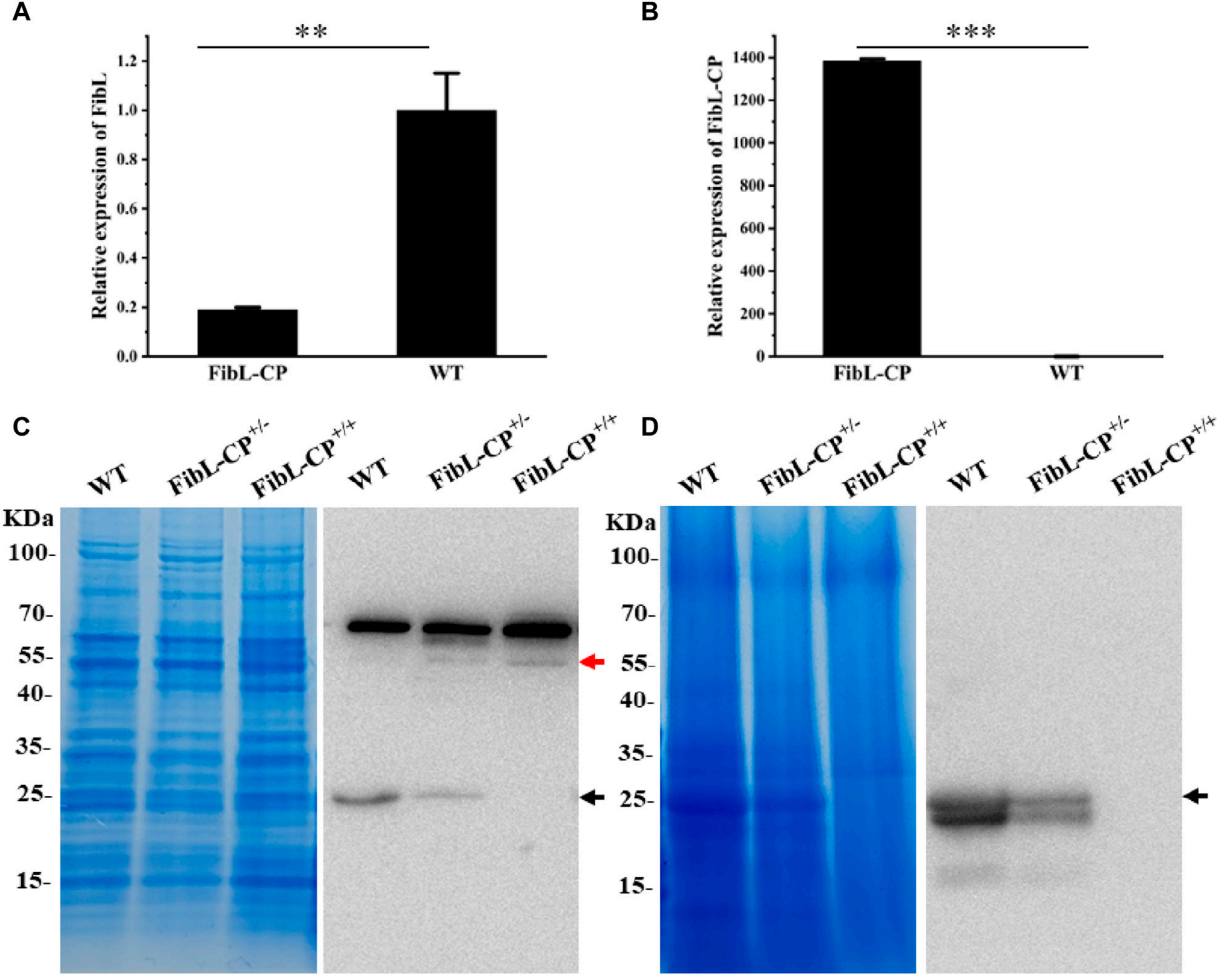
Transcription and expression of FibL–CP. **(A)** Relative transcriptional levels of FibL in FibL–CP and WT strains. **(B)** Relative transcriptional levels of FibL–CP in FibL–CP and WT strains. Three independent biological replicates were used for qRT-PCR, and results are expressed as the mean ± SD. **(C)** SDS–PAGE and western blotting analysis of total protein in the PSG. **(D)** SDS–PAGE and western blotting analysis of total protein in cocoon shells. WT, wild type; FibL–CP^+/−^, heterozygote; FibL–CP^+/+^, homozygote. The black arrow indicates the FibL protein, and the red arrow indicates the FibL–CP protein. In the cocoon shells of WT and FibL-CP^+/−^, there is a smaller band below the FibL protein (26 kDa, black arrow), which we speculated is truncated FibL protein (24.7 KDa, GenBank: AAK13024.1)

FibL has long been of interest as one of the components of silk protein, and FibH, another component of silk protein, has previously been modified by gene editing (Xu et al., 2018; Junpeng et al., 2023; Takasu et al., 2023). However, there has rarely report of site-specific knock-in of FibL by HDR. Here, the CP gene from silkworm was inserted into exon 7 of FibL by TALEN-mediated HDR. The relative mRNA levels of FibL and FibL–CP in the PSG of the FibL–CP transgenic silkworm were determined by qRT-PCR. In FibL–CP animals, abundant expression of the FibL–CP gene was detected, whereas we did not detect any expression of this gene in WT animals (Figure 5B); therefore, endogenous FibL transcriptional levels were markedly decreased in the FibL–CP heterozygous silkworm compared with WT (Figure 5A). Subsequently, we extracted total protein from the PSG of the FibL–CP and WT strains, and detected the expression of FibL–CP protein. The theoretical molecular weight of FibL–CP protein is 57.587 kDa. In the FibL–CP heterozygote, both FibL–CP and FibL proteins were expressed; by contrast, only FibL–CP protein was expressed in the homozygote, and only FibL protein was expressed in the WT animal (Figure 5C). Grayscale analysis of the bands showed that the content of FibL–CP protein was comparable with that of the native FibL in the heterozygote (Supplementary Figure S4). In addition, we extracted total protein from the cocoon shell. SDS–polyacrylamide gel electrophoresis (PAGE) and western blotting analysis revealed that FibL–CP protein failed to be secreted into the cocoon shell (Figure 5D). The homozygote showed an abnormal cocoon shell owing to the absence of FibL (Supplementary Figure S3), with a phenotype similar to that of fibroin-deficient line *Nd-s* ^*D*^ (Takei et al., 1987). These results indicate that the fusion of FibL with CP was successfully transcribed and translated as a new protein in the PSG of FibL–CP strains. Simultaneously, the fusion of CP may have influenced the fibrosis of silk protein and affected spinning and cocooning in FibL–CP strains.

### 3.5 Morphology and mechanical properties of silk fiber

**FIGURE 6:**
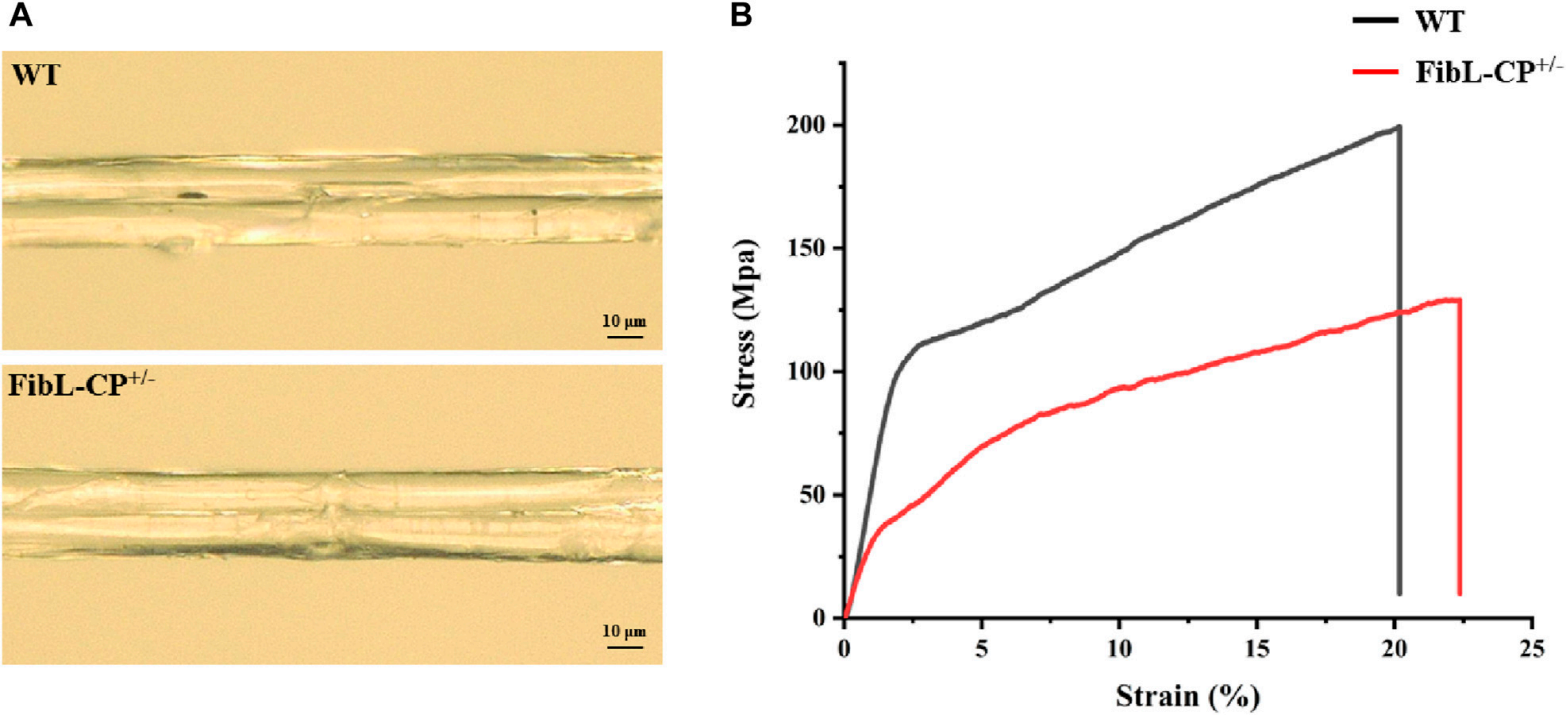
Characteristics of silk fibers. **(A)** Surface of FibL–CP and WT silk fibers. **(B)** Stress–strain curves for FibL–CP and WT silk fibers. The WT is shown in black, and FibL–CP is shown in red.

To further verify the effects of a reduction in FibL levels on silk fiber formation, the silk properties of the FibL–CP heterozygote were measured. The average diameter of FibL–CP silk fibers was 25.9 µm, compared with 25.3 µm for the WT; that is, there was no significant difference between the two strains (Figure 6A). However, the FibL–CP silk fibers had an average breaking strength of 129.1 MPa, compared with 199.4 MPa for the WT, a significant decreased. FibL–CP silk fibers could extend up to 21.9% on average, making them slightly more elastic than their WT counterparts (Figure 6B; Supplementary Table S2). SDS-PAGE and western blotting analysis of proteins from the cocoon shells showed that expression of FibL was reduced in the FibL–CP strain, with no FibL detected at all in the homozygote. The new FibL–CP protein containing complete FibL was not detected in the cocoon shells of all strains (Figure 5D). These results demonstrate the importance of FibL in the formation and spinning of silk fibers (Tanaka et al., 1999; Inoue et al., 2000; Inoue et al., 2004). FibL–CP protein was expressed in the PSG but not secreted into the cocoon shell, possibly owing to either the physicochemical properties of CP or the destruction of FibL.

### 3.6 Prediction of binding between the CTD of FibH and FibL–CP protein

**FIGURE 7:**
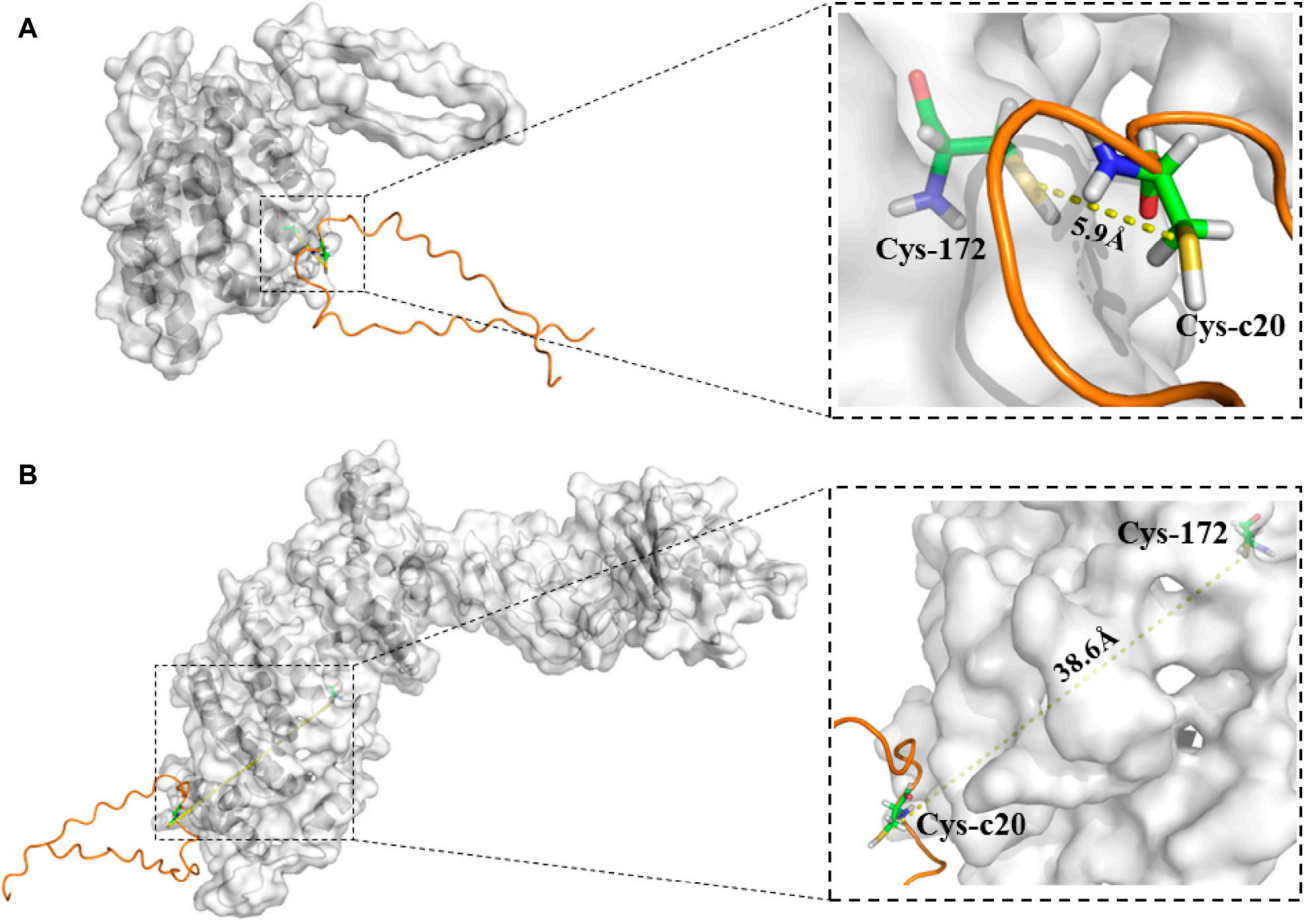
Model of binding of the CTD of FibH to FibL or FibL–CP proteins. **(A)** The CTD of FibH binds to the FibL protein, and Cys-c20 of the FibH-CTD is 5.9 Å away from Cys172 of FibL. **(B)** The CTD of FibH binds to the FibL–CP protein, and Cys-c20 of the FibH-CTD is 38.6 Å away from Cys172 of FibL–CP.

In silkworm, Cys-172 of FibL forms a disulfide bond with Cys-c20 (20th from the C terminus) of FibH, which is required for the intracellular transport and secretion of silk fibroin (Takei et al., 1987; Tanaka et al., 1999). A partial deletion of the FibL gene that caused failure to form the disulfide bond between FibL and FibH resulted in the production of the *Nd-s* ^*D*^ mutant silkworm (Takei et al., 1987). Here, the homozygote of the FibL–CP strain had a similar phenotype to *Nd-s* ^*D*^, and we hypothesized that this could also be owing to disruption of the disulfide bond between FibL–CP and FibH. Hence, we used molecular docking models to predict the disulfide bond between FibL–CP and FibH. All predicted docking models were clustered by the HADDOCK server; the lower the energy, the more stable the binding. The model with highest score from the low-energy cluster was selected for analysis (Supplementary Figure S5). In the model of FibL–CP docking with the CTD of FibH, we observed that the fusion of CP protein prevented the CTD of FibH from entering the region near Cys-172 of the FibL–CP protein, which would lead to a change in the distance between Cys-172 and Cys-c20. We measured the distance between these two cysteine residues and found that it was 5.9 Å in the WT, but 38.6 Å in the FibL–CP docking model (Figure 7). This significant increase in distance may prevent the formation of the disulfide bond between FibL–CP and FibH, resulting in failure of silk fibroin secretion (Tanaka et al., 1999). Hence, we speculated that the impaired secretion of silk fibroin would further reduce the weight and size of the cocoon shells (Figure 3E; Figure 4A).

## 4 Discussion

The development of gene editing has greatly expanded the potential to manipulate the genome of silkworm. Although the *piggy-Bac* transposon-mediated transgene has been established in silkworm (Tamura et al., 2000), and the expression of exogenous proteins by silkworm silk gland bioreactors has been successfully achieved (Tomita et al., 2003; Long et al., 2021; Wu et al., 2021; Chen et al., 2022), the transposon-based systems have defects including random insertion, gene drift, and unrestricted expression of inserted genes, which can cause toxicity to the host (Tomita et al., 2007; Fraser, 2012). The advent of targeted gene editing, including ZNFs, TALEN, and CRISPR/Cas9, has provided new strategies for heterologous gene expression. Although the efficiency of gene targeting transformation mediated by ZNFs or CRISPR/Cas9 is extremely low in silkworm (Takasu et al., 2010; Junpeng et al., 2023), TALEN-mediated transformation shows considerable efficiency and allows for more stable expression and inheritance of exogenous genes (Xu et al., 2018; Li et al., 2021). Therefore, in order to trigger endogenous DSB repair in silkworm, we used TALEN to generate a DSB in the silkworm genome. Using the repair template (donor vector) we provided, HDR repair occurred, and the exogenous gene was site-directed inserted into the genome. As the FibH protein is a major component of fibroin, there was a risk that the targeted insertion of exogenous genes into FibH would affect spinning and cocooning (Xu et al., 2018; Takasu et al., 2023). By contrast, FibL, another component of fibroin, is only inferior to FibH express in silkworm, and the expression of exogenous genes using the native FibL promoter by HDR has been rarely reported. Hence, we selected FibL as the target gene.

In this study, we developed a FibL site-specific insertion system in *B. mori* using TALEN-mediated HDR. We successfully extended the native FibL with CP derived from silkworm. Sanger sequencing showed that the insertion of the CP gene was precise and seamless (Figure 3D). Further qPCR and western blotting analyses showed that the fusion gene was correctly transcribed and translated into a new protein in the PSG of FibL–CP strains (Figure 5), and that the expression level of FibL–CP protein was comparable with that of the native FibL protein. Unfortunately, we found that the homozygote failed to cocoon (Supplementary Figure S3), exhibiting a phenotype similar to that of the fibroin-deficient line *Nd-s* ^*D*^ and FibL-knockout mutant (Takei et al., 1987; Ye et al., 2021). We speculated that this may have been because the insertion of the CP gene affected the formation of the disulfide bond between FibL–CP and FibH, resulting in abnormal secretion of fibroin. Thus, we used molecular docking analysis, in which the CTD of FibH was docked to the FibL–CP protein and FibL, respectively. In the FibL docking model, the distance between Cys-c20 of FibH and Cys-172 of FibL was 5.9 Å, compared with 38.6 Å in FibL–CP (Figure 7). This was a significant increase in distance that would have prevented the formation of a disulfide bond between FibL–CP and FibH, affecting the formation and secretion of silk proteins. Hence, a marked degeneration of the PSG was seen in the homozygote (Supplementary Figure S3), consistent with the phenotype of the FibL-knockout mutant. In previous studies, a truncated FibL lacking Cys-172 showed an inability to form disulfide bonds with FibH, which affected silk protein secretion (Takei et al., 1987). In the present study, complete FibL was fused with CP, which prevented the CTD of FibH from entering the active region to form a disulfide bond with Cys172 of FibL–CP. In addition, tensile tests of FibL–CP heterozygote silk fibers revealed that the silk fiber with decreased FibL levels had weaker strength. These results suggested that FibL is important for silk protein formation and secretion.

Furthermore, the physicochemical properties of the fusion proteins may affect spinning and cocooning. In 2005, Inoue et al. transformed the FibL–GFP open reading frame into *Nd-s* ^*D*^ via a *PiggyBac*-mediated transgene; the transgenic silkworm resumed cocoon formation, and green fluorescence was detected in the cocoon shell (Inoue et al., 2005). It has also been reported that a FibL–eGFP–MaSp1(6) fusion expression transgenic silkworm constructed by CRISPR/Cas9-mediated non-homologous end joining could spin and cocoon normally (Zhang et al., 2019). From these reports, it can be inferred that the strategy of FibL fusion expression is feasible; however, the failure of the FibL–CP homozygote to spin and cocoon may be attributed to the fact that the physicochemical properties of CP are different from those of GFP or MaSp1. CP contains nearly 42% histidine, a characteristic that would probably be detrimental to fibrosis. We selected CP as the insertion protein to fuse with FibL, on the one hand, because CP is derived from silkworm and thus more amenable to expression in that species; on the other hand, because it has the potential to be developed into a biological film material. The failure of the FibL–CP homozygote to spin and cocoon was consistent with a recent study that replaced the heavy chain core region with a highly ordered synthetic repeat sequence resulted in naked pupa in the homozygote (Takasu et al., 2023). However, a transgenic silkworm constructed using the same strategy, in which the spider silk gene (MiSp) replaced the heavy chain core region, was capable of spinning and cocoon and showed good production of transgenic silk (Junpeng et al., 2023). Based on these reports, it can be speculated that the insertion of different genes may differentially affect spinning and cocooning, owing to differences in the proteins encoded by the genes with respect to hydropathicity, pI, stability, and so on. Therefore, the CP protein may not be able to undergo conformation transition and fibrosis in silk glands.

Researchers have used artificial FibL promoters to express exogenous proteins in the PSG of silkworm; however, expression levels were generally low (Tomita et al., 2003; Xu, 2014), probably because endogenous FibL expression competitively inhibits the expression of exogenous genes, and the truncated artificial promoter is not as efficient as the endogenous FibL promoter. These drawbacks can be avoided by using endogenous-promoter-mediated gene expression (Xu et al., 2018). In present study, the FibL–CP fusion protein had a high expression level. Under this bioreactor system, an attempt can be made to add 2A self-cleavage peptide in front of the exogenous protein to achieve non-fusion expression of the exogenous protein. This would be a possibility to improve the expression of non-fusion proteins. The applications of the FibL site-directed fusion system are not limited to protein expression but could include other genetic manipulations, such as targeted insertion of Lox sites and protein tagging. The FibL fusion expression system can also be used to directionally modify the endogenous gene (FibL) of silkworm and provide insights for the improvement of silkworm varieties in the future. Moreover, because the exogenous proteins are tightly combined with the silk fibers and the fibroin protein was insoluble, it is still difficult to purify the target protein from the total protein of silkworm. But, sericin, another component of silk protein, is soluble. Fusion expression of the exogenous protein with sericin may be beneficial to purification (Li et al., 2021). Therefore, the strategy of fusion expression can also be applied to sericin mediated targeted expression, and sericin is distributed on the surface of silk fibers, which may be more conducive to the extraction of exogenous proteins.

In summary, we have established the native FibL in-fusion expression system by TALEN-mediated HDR in silkworm, and shown that an exogenous gene could be expressed at satisfactory levels in this system. Thus, we have provided a new strategy for the expression of exogenous proteins in the silk gland of silkworm, as well as a new approach for improvement of silk properties. In the future, we will attempt to express other proteins of interest using this system, such as spider silk genes, for silk performance improvement; medical proteins with 2A self-cleavage peptide, etc. And improve the economic value of the silk gland bioreactor.

## Data Availability

The original contributions presented in the study are included in the article/Supplementary Material, further inquiries can be directed to the corresponding author.
