# Supplementary material for: TALEN-mediated homologous-recombination-based fibroin light chain in-fusion expression system in Bombyx mori
Source: Front Bioeng Biotechnol. 2024 May 20;12:1399629. doi: 10.3389/fbioe.2024.1399629 (PMC11144906; doi:10.3389/fbioe.2024.1399629)
Supplement: Supplementary file 1 [file DataSheet1.pdf]

Supplementary Material

TALEN-mediated homologous-recombination-based fibroin light chain in-fusion expression system in *Bombyx mori*

Shihua Yu, Huoqing Zheng, Xiaogang Ye, Xiangping Dai , Xinqiu Wang, Shuo Zhao, Xiaoyan Dai, Boxiong Zhong*

*** Correspondence:** Boxiong Zhong: bxzhong@zju.edu.cn

Contents

Figure S1 Target site 2 was assessed by Sanger sequencing. The yellow background is the binding area, and the lowercase letters are the spacer area. 2

Figure S2 FibL-CP fusion transgenic silkworm 5' junction and 3' junction amplification. M represents DNA marker; 1, 2, 3 represent FibL-CP-positive silkworm, respectively; and WT represents wild type. 2

Figure S3 Observation of cocoon shell and silk gland tissue of transgenic silkworm. The entire silk gland tissue and its corresponding cocoon shell. FibL-CP$^{+/-}$ represents heterozygote; FibL-CP$^{+/+}$ represents homozygote. All the scale bars represent 5 mm. 2

Figure S4 The gray analysis of FibL-CP protein and FibL protein 3

Figure S5 The electrostatics energy of the combined model. (A) The result of binding of the CTD of FibH to the FibL protein, cluster 1 had the lowest energy. (B) The result of binding of the CTD of FibH to the FibL-CP protein, cluster 5 had the lowest energy. 3

Table S1 Primers used in this study. 4

Table S2 Mechanical properties of FibL-CP and WT silk fibers. 4

Supplementary figures

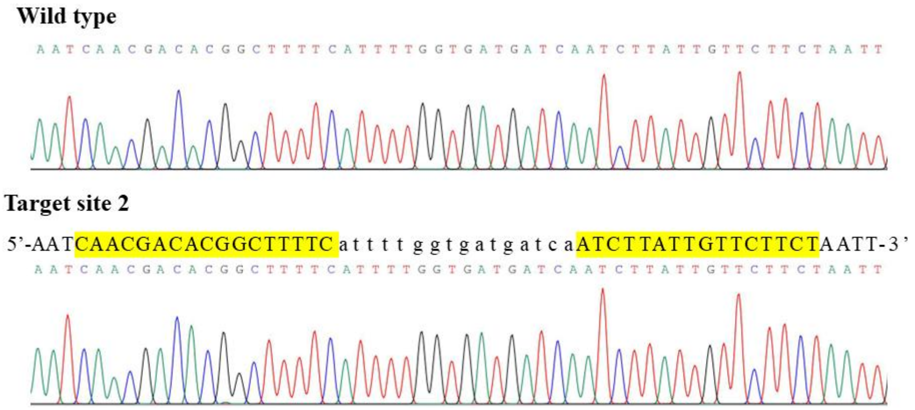

Figure S1 Target site 2 was assessed by Sanger sequencing. The yellow background is the binding area, and the lowercase letters are the spacer area.

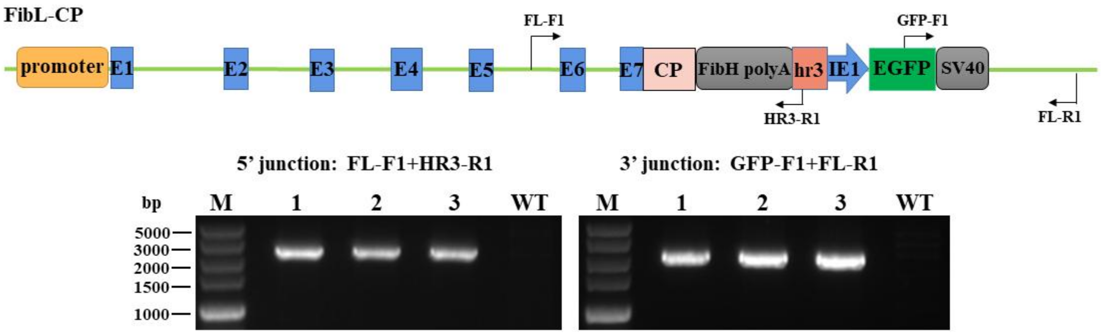

Figure S2 FibL-CP fusion transgenic silkworm 5’ junction and 3’ junction amplification. M represents DNA marker; 1, 2, 3 represent FibL-CP-positive silkworm, respectively; and WT represents wild type.

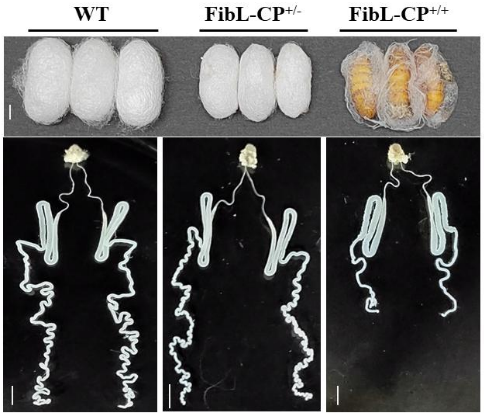

Figure S3 Observation of cocoon shell and silk gland tissue of transgenic silkworm. The entire silk gland tissue and its corresponding cocoon shell. FibL-CP$^{+/-}$ represents heterozygote; FibL-CP$^{+/+}$ represents homozygote. All the scale bars represent 5 mm.

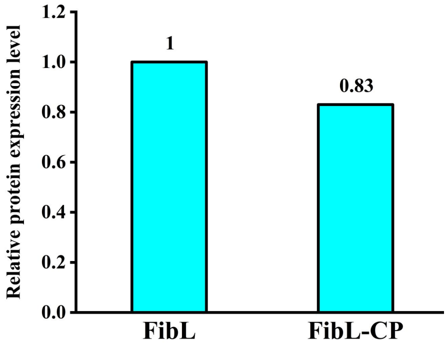

Figure S4 The gray analysis of FibL-CP protein and FibL protein

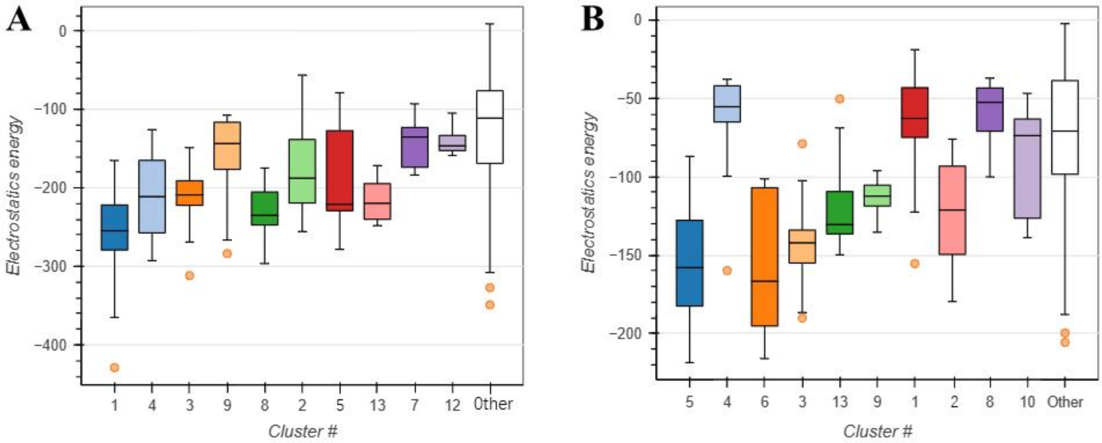

Figure S5 The electrostatics energy of the combined model. (A) The result of binding of the CTD of FibH to the FibL protein, cluster 1 had the lowest energy. (B) The result of binding of the CTD of FibH to the FibL-CP protein, cluster 5 had the lowest energy.

Supplementary table

Table S1 Primers used in this study.

Primer name	Sequence(5′-3′)	Purpose
FL-F1	TGTGGCCAGATGACGTCACAGTGG	5′ junction
HR3-R1	CGCACGATTAAGTATGAATCATAAGC	
GFP-F1	TCTTCAAGTCCGCCATGCCCGAAG	3′ junction
FL-R1	TGACCAGGTCAGTTGGGATAGCATG	
FL-F2	GAAGAGTACTGCATCGTCAAGAGATTG	qRT-PCR
FL-R2	TACGTCTGTGATTGATCCAGCTGAT	
FL-F3	TGAGAGGCGTTGGCAACGGTAAT	qRT-PCR
CP-R1	TGGGTGAGCCATCGCAGCAATA	
GAPDH-F	GAAAAGGGAGCTCAAGTGGTCGC	qRT-PCR
GAPDH-R	CAACAAGGAATCCATCCTGAACCTC	

Table S2 Mechanical properties of FibL-CP and WT silk fibers.

Strains	Maximum stress (Mpa)	Maximum strain (%)	Young's modulus (GPa)	Average diameter (μm)
FibL-CP(n=30) (heterozygote)	129.1±18.7**	21.9±1.8*	3.5±0.5	25.9±1.4
WT (n=30)	199.4±25.9	20.2±2.3	3.3±0.7	25.3±1.9

Data present mean values ± SD
